# Plasma vesicle-associated membrane protein 2 and glial fibrillary acidic protein associate with synaptic density in older adults without dementia

**DOI:** 10.1093/braincomms/fcaf207

**Published:** 2025-05-27

**Authors:** Steffi De Meyer, Soha Alali, Maarten Laroy, Thomas Vande Casteele, Margot Van Cauwenberge, Julie Goossens, Charlotte De Rocker, Jeroen Vanbrabant, Eugeen Vanmechelen, Jan Van den Stock, Filip Bouckaert, Koen Van Laere, Mathieu Vandenbulcke, Louise Emsell, Koen Poesen

**Affiliations:** Laboratory for Molecular Neurobiomarker Research, Department of Neurosciences, Leuven Brain Institute (LBI), KU Leuven, 3000 Leuven, Belgium; Laboratory for Molecular Neurobiomarker Research, Department of Neurosciences, Leuven Brain Institute (LBI), KU Leuven, 3000 Leuven, Belgium; Centre for Neuropsychiatry, Department of Neurosciences, Leuven Brain Institute (LBI), KU Leuven, 3000 Leuven, Belgium; Centre for Neuropsychiatry, Department of Neurosciences, Leuven Brain Institute (LBI), KU Leuven, 3000 Leuven, Belgium; University Psychiatric Centre (UPC), Department of Geriatric Psychiatry, KU Leuven, 3000 Leuven, Belgium; Centre for Neuropsychiatry, Department of Neurosciences, Leuven Brain Institute (LBI), KU Leuven, 3000 Leuven, Belgium; Neurology Department, UZ Leuven, 3000 Leuven, Belgium; ADx NeuroSciences NV, 9052 Ghent, Belgium; ADx NeuroSciences NV, 9052 Ghent, Belgium; ADx NeuroSciences NV, 9052 Ghent, Belgium; ADx NeuroSciences NV, 9052 Ghent, Belgium; Centre for Neuropsychiatry, Department of Neurosciences, Leuven Brain Institute (LBI), KU Leuven, 3000 Leuven, Belgium; University Psychiatric Centre (UPC), Department of Geriatric Psychiatry, KU Leuven, 3000 Leuven, Belgium; Centre for Neuropsychiatry, Department of Neurosciences, Leuven Brain Institute (LBI), KU Leuven, 3000 Leuven, Belgium; University Psychiatric Centre (UPC), Department of Geriatric Psychiatry, KU Leuven, 3000 Leuven, Belgium; Nuclear Medicine and Molecular Imaging, Department of Imaging and Pathology, KU Leuven, 3000 Leuven, Belgium; Division of Nuclear Medicine, UZ Leuven, 3000 Leuven, Belgium; Centre for Neuropsychiatry, Department of Neurosciences, Leuven Brain Institute (LBI), KU Leuven, 3000 Leuven, Belgium; University Psychiatric Centre (UPC), Department of Geriatric Psychiatry, KU Leuven, 3000 Leuven, Belgium; Centre for Neuropsychiatry, Department of Neurosciences, Leuven Brain Institute (LBI), KU Leuven, 3000 Leuven, Belgium; University Psychiatric Centre (UPC), Department of Geriatric Psychiatry, KU Leuven, 3000 Leuven, Belgium; Translational MRI, Department of Imaging and Pathology, KU Leuven, 3000 Leuven, Belgium; Laboratory for Molecular Neurobiomarker Research, Department of Neurosciences, Leuven Brain Institute (LBI), KU Leuven, 3000 Leuven, Belgium; Laboratory Medicine, UZ Leuven, 3000 Leuven, Belgium

**Keywords:** blood biomarkers, synapse pathology, VAMP2, SNAP25, SV2A PET

## Abstract

Synaptic loss is an early hallmark of many neurological disorders, including Alzheimer’s disease, but also occurs in aging brains as evidenced by PET- and CSF-based biomarker studies. This cross-sectional study investigates how blood-based synaptic proteins and other biomarkers relate to synaptic density in brains of older adults without dementia, and how these associations are mediated by gray matter (GM) loss. Plasma levels of synaptic biomarkers including synaptosomal-associated protein of 25 kDa and vesicle-associated membrane protein 2 as well as amyloid, tau, neurodegeneration and neuroinflammation (ATN(I)) biomarkers including the amyloid-β_1-42_/amyloid-β_1-40_ ratio, phosphorylated tau181, neurofilament light and glial fibrillary acidic protein were quantified in 61 older adults without dementia [mean age ± standard deviation = 71 ± 6 years, median Mini-Mental State Examination score (interquartile range) = 29 (3), 64% female, 38% late-life depression] who underwent synaptic vesicle glycoprotein 2A PET and T_1_-weighted MRI. Subsets underwent amyloid (*n* = 49) and tau (*n* = 52) PET. The study population demonstrated limited PET-based amyloid-β and tau pathology, which did not associate with any of the investigated plasma biomarkers. Synaptic plasma biomarkers correlated with each other [Spearman’s *ρ* (95% confidence interval) 0.37 (0.14–0.57), *P* = 0.019], but not with the ATN(I) plasma biomarkers. Plasma vesicle-associated membrane protein 2 associated with synaptic vesicle glycoprotein 2A PET within frontal, temporal, and occipital cortices [*β*_s_ (95% confidence interval) −0.75 (−1.06 to −0.44)], independent of age. Glial fibrillary acidic protein also demonstrated associations with synaptic vesicle glycoprotein 2A PET, particularly within temporal regions, the cingulate gyrus, and the precuneus [*β*_s_ (95% confidence interval) −0.30 (−0.48 to −0.12)] when corrected for age and additionally within the caudate nucleus and thalamus when not [*β*_s_ (95% confidence interval) −0.45 (−0.64 to −0.25)]. Similar associations were found in subgroups without, respectively, Alzheimer’s disease pathology, or late-life depression (with cognitive impairment). No associations with synaptic vesicle glycoprotein 2A PET were found for synaptosomal-associated protein of 25 kDa, phosphorylated tau181, neurofilament light or amyloid-β_1-42_/amyloid-β_1-40_, likely due to the minimal presence of Alzheimer’s disease pathology in the study population. GM volume associated with glial fibrillary acidic protein [*β*_s_ (95% confidence interval) −0.15 (−0.25 to −0.05)] and partially (29%) mediated its association with synaptic vesicle glycoprotein 2A PET. In conclusion, plasma levels of glial fibrillary acidic protein and vesicle-associated membrane protein 2 may reflect synapse pathology independent of age and beyond general neuronal loss, even in absence of detectable Alzheimer’s disease pathology.

## Teaser

De Meyer *et al*. report that vesicle-associated membrane protein 2 and glial fibrillary acidic protein levels in plasma reflect brain synaptic density—as indicated by synaptic vesicle glycoprotein 2A PET—in individuals without dementia or underlying Alzheimer’s disease pathology and independent of neurodegeneration.

## Graphical Abstract

**Graphical Abstract:**
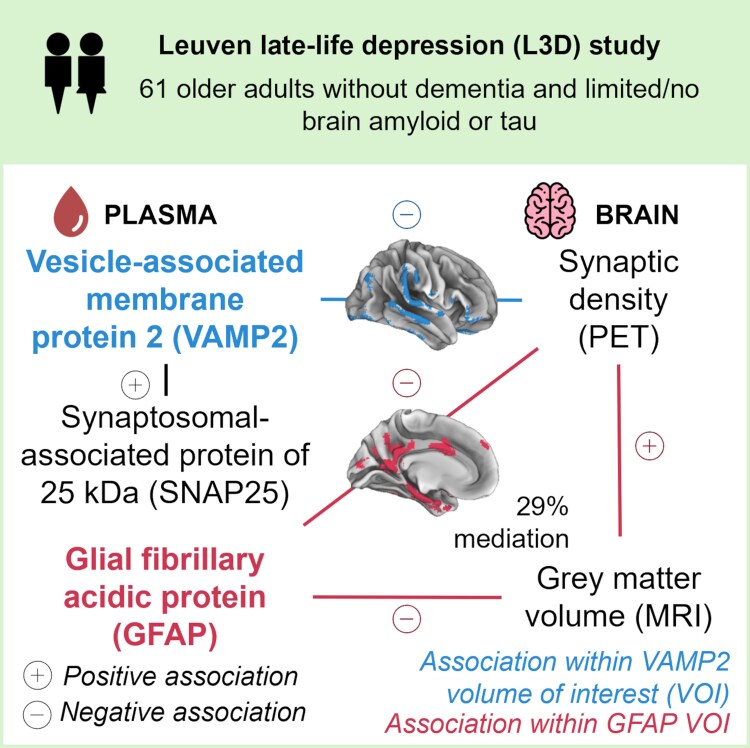

## Introduction

Synapses are the vital junctions for neuronal communication, ensuring a smooth information flow throughout the nervous system. Minor dysfunctions at these synapses can impair cognitive function and occur in normal aging as well as a variety of neurological disorders.^1,2^ At early stages, the reduction in brain connectivity due to synapse pathology (i.e. loss or dysfunction) may be subtle, with compensatory responses balancing the impact on brain function.^3-5^ Age-related synaptic loss is typically mild and progresses slowly without inducing substantial neuronal loss.^6-8^ In Alzheimer’s disease,^9-12^ but also other neurodegenerative disorders, including frontotemporal dementia,^13^ Lewy body dementia,^14^ Parkinson’s disease,^15^ and progressive supranuclear palsy,^16^ synaptic loss is more prominent^6,17^ and constitutes one of the earliest pathological signs preceding neuronal loss, as indicated by rodent^9,10,13,15^ and human neuropathology^11,12,14,16^ studies.

These insights have been corroborated by biomarker studies, in which quantification of synapse pathology has so far predominantly relied on PET- or CSF-based measures. [^11^C]UCB-J PET, for example, targets synaptic vesicle glycoprotein 2A (SV2A), a presynaptic protein that is ubiquitously expressed in most synaptic vesicles in the human brain, thus serving as a proxy for synaptic density.^18,19^ A recent neuropathological study reported moderate to strong correlations between SV2A and synaptophysin densities across multiple cortical brain regions and neurodegenerative disorders (i.e. Alzheimer’s disease, progressive supranuclear palsy, and frontotemporal lobar degeneration with TAR DNA-binding protein 43 inclusions) supporting its validity as a surrogate marker for synaptic density.^20^ Reduced [^11^C]UCB-J uptake has been observed in healthy aging, albeit mostly limited to the caudate nucleus,^21,22^ as well as in distinct brain regions in Alzheimer’s disease,^23,24^ Lewy body dementia,^25,26^ Parkinson’s disease,^25,27,28^ frontotemporal dementia and progressive supranuclear palsy,^29^ and this with a magnitude greater than that of gray matter (GM) atrophy,^26,30^ further suggesting that synaptic loss precedes neuronal loss. CSF levels of pre- and postsynaptic proteins also become altered in a variety of neurodegenerative diseases, often manifesting even in preclinical stages.^31-38^ Distinct disease-dependent patterns of CSF synaptic markers have been observed. For instance, changes in CSF levels of the presynaptic synaptosomal-associated protein of 25 kDa (SNAP25)^31-33,35-37,39-42^ and vesicle-associated membrane protein 2 (VAMP2) proteins^31,36,38,39^ have been reported in Alzheimer’s disease, frontotemporal dementia and/or Lewy body dementia compared with controls, whereas CSF-based changes in the presynaptic protein β-synuclein,^41,43^ the post-synaptic protein neurogranin,^31,32,35-37,40-43^ as well as in neuregulin-1^44,45^ have been primarily observed in Alzheimer’s disease. SNAP25 and VAMP2 are both part of the soluble N-ethylmaleimide-sensitive factor attachment protein receptor (SNARE) complex that is situated on pre-synaptic secretory vesicles and mediates neurotransmitter release into the synaptic cleft.^46^

Given the early involvement of synapses in neurodegenerative disorders and their close links to neuropathology and cognitive decline, synaptic proteins present promising targets for monitoring in therapeutic trials. Consequently, there is a growing interest in identifying blood-based synaptic biomarkers to facilitate such large-scale and repeated sampling. However, not all synaptic proteins exhibit biomarker potential in blood. Although detectable in blood, the peripheral expression of neurogranin makes it challenging to determine to which extent blood-based neurogranin changes reflect changes in the brain.^47^ In contrast, β-synuclein expression is specific to the central nervous system^48^ and elevated in blood of Alzheimer’s disease patients, even from preclinical stages, in which it correlates with Alzheimer’s disease pathology, neurodegeneration, and cognitive performance.^49-52^ Despite peripheral expression sources, elevated blood-based neuregulin-1 levels have also been observed in Alzheimer’s disease patients compared with controls.^53^ Recently, immunoassays have been developed for quantification of SNAP25 and VAMP2 in plasma. The extent to which blood levels of these synaptic proteins reflect synaptic changes in the brain occurring in normal aging or at the onset of neurodegenerative diseases remains unexplored. We aimed to answer these questions by examining whether blood-based levels of VAMP2 and SNAP25 along with blood-based ATN(I) biomarkers are associated with SV2A PET in the brains of older adults without dementia. In this study, amyloid-β (Aβ)_1-42_/Aβ_1-40_, phosphorylated tau (pTau)181 and neurofilament light (NfL) represented the ‘A’, ‘T’ and ‘N’ markers reflecting amyloid and tau pathology, and neurodegeneration, respectively. Glial fibrillary acidic protein (GFAP) represented the ‘I’ marker reflecting neuroinflammation.^54,55^ We also evaluated whether the associations of these blood-based biomarkers with SV2A PET were age-dependent or driven by general GM loss, as quantified by MRI.

## Materials and methods

### Study population

The study population in this cross-sectional study consisted of 61 older adults without dementia selected from the Leuven late-life depression (L3D) study based on the availability of a baseline plasma sample, SV2A PET and T_1_-weighted structural MRI.^56^ Subsets of the study population additionally underwent amyloid (*n* = 49) and tau (*n* = 52) PET. Out of the 61 participants from the L3D study, 38 were cognitively unimpaired older adults recruited from the local community with test scores on clinical and neuropsychological examinations within published norms and without prior or current depressive illness. The other 23 participants, who were matched for age and sex, were diagnosed with late-life depression according to the diagnostic and statistical manual of mental disorders 5 criteria using the MINI-Plus^57^ at the University Psychiatric Centre of KU Leuven in Belgium. All patients with late-life depression demonstrated some degree of cognitive impairment but did not have a known prior diagnosis of dementia at the time of study inclusion. They were all undergoing treatment for depression (with anticholinergics, antidepressants, benzodiazepines, antipsychotics or opiates, see Supplementary Table 1) and demonstrated stable major depressive disorder at time of study inclusion. No differences in plasma biomarker levels were found between treated and untreated groups. Use of illicit drugs was an exclusion criterion of the L3D study. For both subcohorts, selection criteria included age 60 years or older and no evidence of a major neurological disorder. An extensive list of inclusion/exclusion criteria was previously published.^56^ Note that, as part of the L3D protocol, a subset of late-life depression patients underwent electroconvulsive therapy (ECT), of whom four were included in the current study. However, all plasma samples and other measures included in the current study were obtained prior to the start of ECT. For all participants, plasma was sampled in the morning after overnight fasting between 6 January 2019 and 28 March 2023, at a median time interval of 0 [interquartile range (IQR) 0–6] days from last cognitive assessment. Imaging examinations took place between 4 July 2019 and 25 October 2023, at a median time interval of 24 (IQR 8–60) days from plasma sampling and 20 (IQR 8–56) days from last cognitive assessment.

### Ethics

Written informed consent was obtained from all participants in accordance with the Declaration of Helsinki, and all study procedures were approved by the Ethics Committee of University Hospitals Leuven (S61968).

### Imaging

SV2A and tau PET scans were acquired on a 3T Signa PET/MR scanner (GE Healthcare, Milwaukee, WI, USA) with simultaneous MRI acquisition using a T_1_-weighted 3D BRAVO sequence. Amyloid PET was acquired on a 16-slice Biograph PET/CT scanner (Siemens, Erlangen, Germany). All tracers were injected through a venous catheter line in the participant’s forearm. For SV2A PET, a [^11^C]UCB-J tracer was injected and PET measurements were acquired in the 60- to 90-min post-injection window. For tau and amyloid PET, [^18^F]MK6240 and [^18^F]flutemetamol, respectively, were injected and PET measurements were acquired in the 90- to 120-min post-injection window. PET data were reconstructed into six frames of 5 min using an ordered subsets expectation maximization iterative (4 iterations and 28 subsets) algorithm and corrected for dead time, randoms and scatter. Attenuation correction was performed using a zero echo time (ZTE) MR scan. Image processing was fully automated using in-house scripts (https://github.com/THOMVDC/PSYPET) written in Matlab based on Statistical Parametric Mapping 12 (SPM12, Wellcome Trust Centre for Neuroimaging, London, UK). Reconstructed [^18^F]MK6240 and [^11^C]UCB-J PET data were corrected for partial volume effects (PVE) using a region-based voxelwise PVE correction algorithm.^58^ Standardized uptake value ratio (SUVR) parametric images representing tracer binding were calculated for [^11^C]UCB-J, [^18^F]MK6240 and [^18^F]flutemetamol using the centrum semiovale as reference region for [^11^C]UCB-J and the cerebellar GM as reference region for both [^18^F]MK6240 and [^18^F]flutemetamol in the participant-specific space.^59^ White matter lesions extracted using a deep-learning based approach applied to 3D FLAIR and T_1_-weighted MR images^60^ were excluded from the centrum semiovale reference region. T_1_-weighted MR images and coregistered PET SUVR images were then normalized to Montreal Neurological Institute space for processing in SPM12. Using these normalized SUVR images, global Aβ load was calculated in a composite volume of interest (VOI) encompassing Aβ-vulnerable regions,^61,62^ and a global measure for tau load was derived from an early tau-vulnerable composite VOI.^63^ Participants were considered to be Aβ-positive if the [^18^F]flutemetamol SUVR in the Aβ-vulnerable VOI exceeded 1.38.^64^ Prior to voxelwise analyses, the normalized [^11^C]UCB-J SUVR maps were smoothed with an 8 mm Gaussian kernel.

Normalized MRI images were segmented using the Computational Anatomy Toolbox 12 within SPM12, and the total intracranial volume (TIV) was calculated. GM volume estimates within biomarker-specific VOIs were extracted from modulated GM maps with a GM intensity threshold > 0.3. These GM volume estimates were normalized through division by the TIV and subsequently scaled through multiplication by 1000 so that its magnitude was comparable to that of other variables in statistical models.

### Plasma measurements

After blood collection, samples were stored at room temperature for 30 min, followed by centrifugation for 10 min at 4°C. The supernatant was then aliquoted into 800 µL aliquots and stored at −80°C. For each participant, one aliquot was shipped to ADx NeuroSciences NV (Ghent, Belgium) for plasma biomarker measurements. Prior to analyses, plasma samples were thawed, vortexed and centrifuged at 10 000 *g* and 4°C for 8 min to remove debris. Sample randomization across runs was performed using the R Package *WPM*. Plasma levels of SNAP25 and VAMP2 were measured using prototype homebrew Simoa assays developed by ADx Neurosciences NV in triplicate and duplicate, respectively. These prototype assays were partially validated according to Andreasson *et al*.^65^ (Supplementary Methods). In the context of this proof-of-concept study, these partial validation characteristics are considered adequate for homebrew assays. However, they are prototype assays that are still in development and further optimization of parameters such as diluents and analyte stability is required. Plasma pTau181 levels were measured in duplicate using the homebrew ADx252-based Simoa assay developed by ADx NeuroSciences NV, as described.^66^ Plasma GFAP, NfL, Aβ_1-42_ and Aβ_1-40_ were quantified in singlicate using the Neurology 4-Plex E Simoa kit (Cat No. 103670, Quanterix, Billerica, MA, USA) according to manufacturer’s instructions. For SNAP25 (*n* = 6) and VAMP2 (*n* = 2), reruns were conducted for samples that exhibited intra-assay coefficients of variation (CV) >20% (SNAP25), as well as for those with single values (VAMP2). In cases where one of the SNAP25 triplicate measurements deviated from the other two (*n* = 8), it was excluded from concentration calculation. For pTau181, eight measurements were below the lower limit of quantification and were therefore assigned a concentration equal to half of the lowest calibration point (1.95 pg/mL). Nine duplicate pTau181 measurements yielded CVs > 20% and were therefore excluded. For an additional 2 samples, no pTau181 measurements were available due to an operational error or insufficient sample volume, thus yielding a total of 50 participants with pTau181 measurements.

### Statistics

Statistical analysis was performed using R software, version 4.2.2 (Foundation for Statistical Computing). Normality was assessed using Shapiro–Wilk tests. Correlations among plasma biomarkers as well as between plasma biomarkers and other continuous variables were calculated using Spearman correlations. Associations between plasma biomarkers and binary or categorical variables were evaluated with respectively Mann–Whitney U-tests and Kruskal–Wallis tests.

As a primary outcome analysis, we evaluated the association between plasma biomarkers and synaptic density using voxelwise multiple linear regression models including SV2A PET SUVR images as outcome and plasma biomarkers as predictor, corrected for confounders. Selection of confounders followed the disjunctive cause criterion and included age and sex as they were previously reported to affect synaptic density, as well as levels of several blood-based biomarkers.^21,31,67-73^ Additionally, a diagnosis of late-life depression was included to account for potential bias introduced by the heterogeneity of the study population, given that 38% of participants was diagnosed with late-life depression. While a previous study in the L3D cohort did not find altered SV2A PET measures in late-life depression patients,^74^ an independent study in a smaller sample of unmedicated younger major depressive disorder patients reported decreased SV2A PET signal.^75^ The significance threshold of voxelwise analyses was set at a cluster-level whole-brain family-wise error (FWE) threshold of *P*_FWE_ < 0.05 with voxel-level set at *P*_uncorrected_ < 0.05. If significant clusters were identified, they were used as biomarker-specific VOIs from which the average (composite) SV2A SUVR value (SUVR_comp_) was extracted. The biomarker-specific SV2A SUVR_comp_ value was used as outcome variable in a simple linear regression model with the respective plasma biomarker as sole predictor. Prior to inclusion of plasma biomarker values in regression models, they were converted to *z*-scores (based on the mean and SD within the Aβ-negative subset) to allow inter-biomarker comparison of effect sizes. Plasma biomarker values were transformed if needed to meet model assumptions. Voxelwise models for the prediction of SV2A PET SUVRs were also constructed without correction for age to investigate the association of plasma biomarkers with age-dependent synaptic loss as well as with age as the sole predictor.

As a secondary outcome analysis, we evaluated whether potential associations between synaptic density (SV2A PET) and plasma biomarker levels were driven by overall neurodegeneration (GM volume). We therefore first constructed linear regression models using GM volume as predictor and plasma biomarkers as outcome. If a significant association was found, we then performed mediation analyses to estimate natural indirect and direct effects using structural equation modelling in R (*lavaan* package), in which SV2A PET was used as predictor, the plasma biomarker was used as the outcome and GM volume as the mediator. Age and sex were included as confounders for the effect of the mediator on the outcome variable.

Lastly, sensitivity analyses were performed to assess whether the observed associations were driven by underlying Alzheimer’s disease pathology or a late-life depression diagnosis (with cognitive impairment). To this end, the regression and mediation analyses were repeated within the study population subsets that were amyloid PET negative (*n* = 45) and did not have a diagnosis of late-life depression (*n* = 38), respectively. The 95% confidence intervals (CIs) for Spearman correlation coefficients as well as direct and indirect effects derived from mediation analyses were calculated using bootstrapping (*n* = 1000). Reported *P*-values were corrected for multiple comparisons using Bonferroni correction, unless specified otherwise, and *P* < 0.05 were considered significant.

## Results

### Cohort demographics and biomarker data

The characteristics of the 61 older adults without dementia from the L3D cohort are presented in Table 1. The study population demonstrated low levels of amyloid PET load [median (IQR) SUVR = 1.20 (0.08), Supplementary Fig. 1A], except for four amyloid PET positive individuals (SUVR > 1.38), as well as low overall tau PET load (mean SUVR ± SD = 0.96 ± 0.15, Supplementary Fig. 1B). No association of sex, *APOE-ɛ4* carrier status, or education level was observed with any of the investigated plasma biomarkers (Supplementary Table 2). GFAP [*ρ* = 0.52 (95% CI 0.31–0.70), *P* < 0.0001], NfL [*ρ* = 0.53 (95% CI 0.29–0.71), *P* < 0.0001], and pTau181 [*ρ* = 0.53 (95% CI 0.28–0.72), *P* = 0.00048] correlated with age, whereas VAMP2, SNAP25, and the Aβ_1-42_/Aβ_1-40_ ratio did not. None of the synaptic markers nor the ATN(I) biomarkers demonstrated associations with brain amyloid or tau PET load (Supplementary Table 2).

**Table 1:** Characteristics of the L3D cohort

Characteristics	*N*	L3D
Female, no. (%)	61	39 (64)
Age, years, mean ± SD	61	71 ± 6
Highest attained level of education (1/2/3/4)^a^, no.	61	7/30/18/6
MMSE/30, median [IQR]	61	29 [3]
*APOE-ɛ4* carriers, no. (%)	61	10 (16)
Depression diagnosis, no. (%)	61	23 (38)
Aβ load, SUVR, median [IQR]	49	1.20 [0.08]
Aβ positive, no. (%)	49	4 (8)
Tau load, SUVR, mean ± SD	52	0.96 ± 0.15
Plasma Aβ_1-42_/Aβ_1-40_, mean ± SD	61	0.065 ± 0.013
Plasma pTau181, pg/mL, median [IQR]	50	12.8 [12.3]
Plasma GFAP, pg/mL, mean ± SD	61	108 ± 46
Plasma NfL, pg/mL, median [IQR]	61	17.9 [11.1]
Plasma VAMP2, pg/mL, median [IQR]	61	47 [32]
Plasma SNAP25, pg/mL, median [IQR]	61	0.91 [0.27]

Continuous data are expressed as mean ± SD when normally distributed and median [IQR] when not. Categorical data are expressed as number (%).

^a^The highest attained levels of education were defined as follows: 1 = primary education, 2 = secondary education, 3 = higher education, 4 = university.

### Correlations between plasma biomarker levels

**Figure 1:**
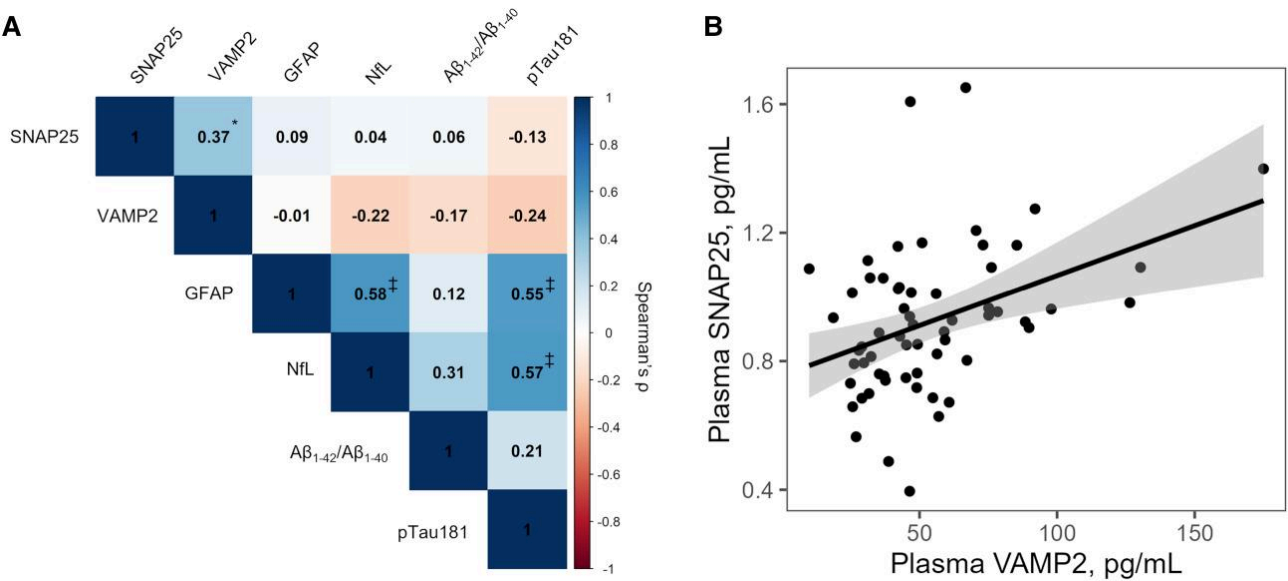
**The correlations between synaptic and ATN(I) biomarkers in plasma in older adults without dementia.** (**A**) Correlation matrix demonstrates the correlations between plasma biomarkers in the L3D cohort (*n* = 61). The colour scale represents the strength and the direction of the Spearman correlation coefficients. (**B**) Scatter plot visualizes the association between the synaptic plasma biomarkers SNAP25 and VAMP2. Each data point represents an L3D study participant. The linear fit (black line) with 95% CI (shaded area), as derived from simple linear regression models using plasma VAMP2 as predictor and SNAP25 as outcome, is superimposed. *P < 0.05; ^‡^*P* < 0.0001 after Bonferroni correction.

Plasma levels of the synaptic markers SNAP25 and VAMP2 moderately correlated with each other [*ρ* = 0.37 (95% CI 0.14–0.57), *P* = 0.019] but did not correlate with any of the ATN(I) plasma biomarkers (Fig. 1A and B). Among the ATN(I) plasma biomarkers, GFAP and NfL correlated with each other [*ρ* = 0.58 (95% CI 0.38–0.74), *P* < 0.0001] and both correlated with pTau181 [*ρ* = 0.55 (95% CI 0.30–0.74), *P* < 0.0001 for GFAP, *ρ* = 0.57 (95% CI 0.32–0.74), *P* < 0.0001 for NfL, Fig. 1A, Supplementary Table 3].

### Associations between plasma biomarkers and brain PET measures of synaptic density

**Figure 2:**
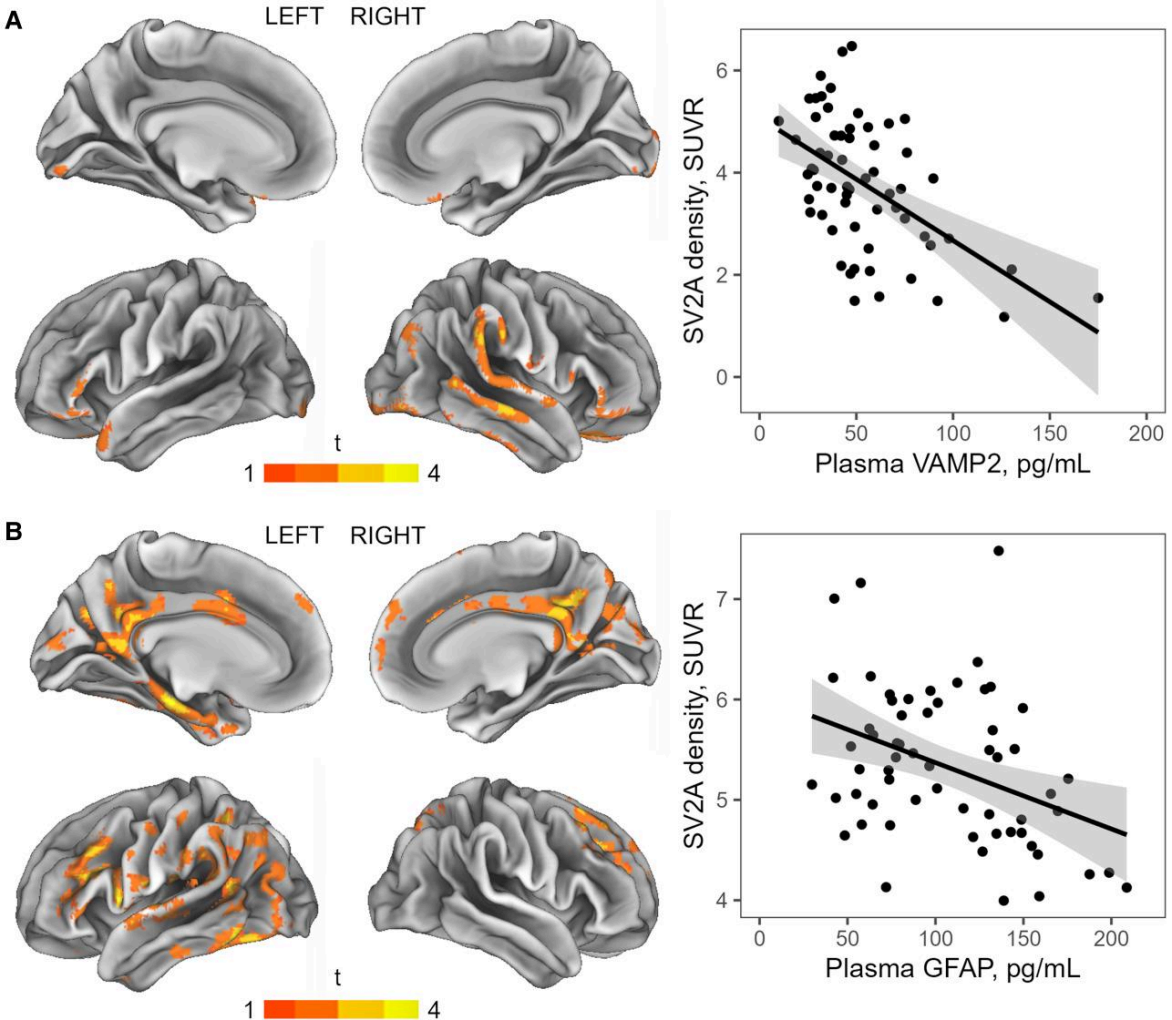
**Associations of plasma GFAP and VAMP2 with synaptic density in the brains of older adults without dementia.** Parametric *T* maps of the age-, sex- and depression-adjusted regional associations between synaptic density and plasma VAMP2 (**A**) or GFAP (**B**) concentrations calculated using voxelwise multiple linear regression models are shown for the L3D cohort (*n* = 61). The significance threshold was set at a whole-brain cluster-level FWE threshold of *P*_FWE_ < 0.05 and an uncorrected voxel-level threshold of *P*_uncorrected_ < 0.05. Thresholded maps were superimposed on the left and right hemisphere of the PALS cortical surface (PALS-B12) using CARET v5.65.^76^ Clusters showing significant associations (shown in the left panels) were used as biomarker-specific VOIs, in which the association strength between plasma biomarkers and synaptic density was quantified by means of the standardized regression coefficient from simple linear models (biomarker as sole predictor, SV2A PET SUVR as outcome). The right panels show the scatterplots of the association between synaptic density and plasma VAMP2 (**A**) or GFAP (**B**) in the respective identified VOIs. Each data point represents an L3D study participant. Linear fits (black lines) with 95% CI (shaded areas), as derived from the respective simple linear regression models, are superimposed. Note that raw biomarker values were plotted to ease interpretation, but statistical analyses were performed on scaled biomarker values (*z*-scores) to enable direct comparison of effect sizes.

Voxelwise analyses adjusted for age, sex and late-life depression diagnosis showed that VAMP2 levels in plasma associated with SV2A PET SUVRs within temporal and frontal brain regions as well as within a small region in the occipital cortex [SUVR_comp,VAMP2_ (mean ± SD) = 3.78 ± 1.31, *β*_s_ = −0.75 (95% CI −1.06 to −0.44), *P* < 0.0001, Fig. 2A]. Within these regions, plasma VAMP2 levels explained 28% of the variance observed in SV2A PET SUVRs [*F*(1,59) = 23.26, *R*^2^ = 0.28]. Plasma GFAP levels associated with SV2A PET SUVRs within temporal regions in addition to the precuneus and cingulate gyrus [SUVR_comp,GFAP_ (mean ± SD) = 5.32 ± 0.76, *β*_s_ = −0.30 (95% CI −0.48 to −0.12), *P* = 0.0048, Fig. 2B], in which they explained 16% of the observed variance in SV2A PET SUVRs [*F*(1,59) = 10.94, *R*^2^ = 0.16]. For NfL, associations with SV2A PET did not survive correction for multiple comparisons [SUVR_comp,NfL_ (mean ± SD) = 4.88 ± 0.65, *β*_s_ = −0.17 (95% CI −0.33 to −0.01), *P* = 0.11, *R*^2^ = 0.07, *F*(1,59) = 4.61, Supplementary Fig. 2]. For plasma levels of SNAP25, pTau181 and Aβ_1-42_/Aβ_1-40_, no clusters demonstrating significant associations with SV2A PET were found. In order to investigate to which extent blood biomarker levels reflect age-dependent synaptic loss, we repeated voxelwise regression analyses without adjustment for age. This revealed associations between VAMP2 and SV2A PET in the same regions as observed for age-independent relationships, yet now bilaterally rather than predominantly in the right hemisphere [*β*_s_ = −0.62 (95% CI −0.89 to −0.36), *P* < 0.0001, Supplementary Fig. 3A]. When not corrected for age, GFAP levels associated with SV2A PET within the thalamus, caudate nucleus and frontal regions in addition to the age-independent associations (i.e. in temporal regions, precuneus and cingulate gyrus) described earlier [*β*_s_ = −0.45 (95% CI −0.64 to −0.25), *P* < 0.0001, Supplementary Fig. 3B]. When using age as the sole predictor for SV2A PET SUVRs, negative associations were observed within the caudate nucleus, thalamus, hippocampus, and frontal regions in both hemispheres (Supplementary Fig. 4).

### Mediating role of neurodegeneration

**Figure 3:**
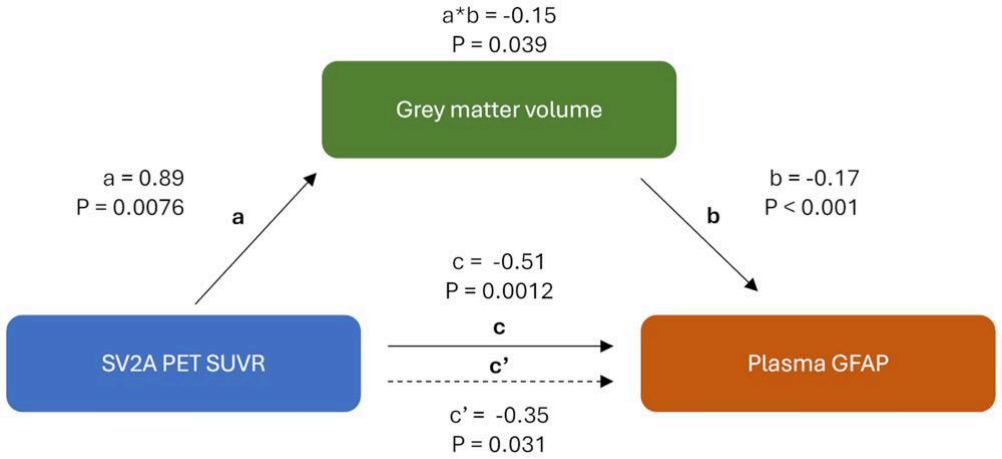
**Mediation model relating GFAP, GM volume and synaptic density.** The mediation model contains SV2A PET SUVRs in the biomarker-specific VOI (as measured by [^11^C]UCB-J PET) as a predictor, GM volume in the biomarker-specific VOI as a mediator and plasma GFAP as outcome in the L3D cohort (*n* = 61). Natural indirect and direct effects were estimated using structural equation modelling (R package *lavaan*). The total effect (*c*) represents the effect of synaptic density on plasma GFAP levels both directly (*c*′) and indirectly through the mediator (ACME = *a* × *b*). Effect sizes of scaled plasma biomarker concentrations (*z*-scores) are shown. GM volume was normalized through division by the TIV and subsequently scaled through multiplication by 1000 so that its magnitude was comparable to that of the other variables. Path weights and corresponding *P-*values obtained through bootstrapping (*n* = 1000) are superimposed.

GM volume in the biomarker-specific VOI (where significant associations between GFAP and SV2A PET SUVRs were found) was associated with plasma GFAP levels [*β*_s_ = −0.15 (95% CI −0.25 to −0.05), *P* = 0.0077]. No association between GM volume and VAMP2 was found (Supplementary Table 4). To determine whether synaptic loss alters blood GFAP levels beyond the neurodegeneration-associated GFAP changes, mediation analysis was performed. This revealed that SV2A PET SUVRs had a direct effect on plasma GFAP levels, independent of GM volume [*c*′ = −0.35 (95% CI −0.70 to −0.05), *P* = 0.031, Fig. 3]. However, GM volume did have a partially mediating role [average causal mediation effect (ACME) = −0.15 (−0.33 to −0.03), *P* = 0.039, 29% mediation].

### Sensitivity analysis

Within the amyloid PET negative subset (*n* = 45, Supplementary Table 5), both VAMP2 and GFAP retained its predictive value for SV2A PET SUVR_comp_ [*β*_s_ = −0.71 (95% CI −1.03 to −0.38) for VAMP2 and *β*_s_ = −0.35 (95% CI −0.55 to −0.14) for GFAP]. Moreover, GFAP demonstrated comparable associations to GM volume [*β*_s_ = −0.16 (95% CI −0.27 to −0.04)] as in the total study population (Supplementary Table 6).

Similarly, when repeating analyses upon exclusion of late-life depression patients (*n* = 38, Supplementary Table 7), the associations of plasma VAMP2 [*β*_s_ = −0.75 (95% CI −1.09 to −0.41)] and GFAP [*β*_s_ = −0.35 (95% CI −0.57 to −0.12)] with SV2A PET SUVR_comp_ values were comparable. The effect size of the association between GFAP and GM volume was also similar but was no longer significant after correction for multiple comparisons [*β*_s_ = −0.14 (95% CI −0.26 to −0.01), Supplementary Table 8].

The size of the ACME of GM volume in the association between SV2A PET and plasma GFAP was comparable to that of the total study population in both the amyloid-negative subset [ACME = −0.11 (−0.24 to −0.003), 19% mediation, *P* = 0.080, Supplementary Fig. 5A] and the subset without late-life depression diagnosis [ACME = −0.14 (−0.38 to 0.01), 25% mediation, *P* = 0.17, Supplementary Fig. 5B], but did not reach significance.

## Discussion

In the present study, we evaluated the associations of blood-based synaptic and ATN(I) biomarkers with synaptic density, measured using SV2A PET, in the brains of older adults without dementia. We found that plasma levels of the synaptic marker VAMP2 were associated with synaptic density in temporal, frontal and occipital cortices, whereas plasma levels of the astrocytic marker GFAP were associated with synaptic density in the temporal cortex, cingulate gyrus and precuneus, even in absence of detectable amyloid or tau pathology. Plasma GFAP, but not VAMP2, was associated with age and, when not accounting for age, the association of GFAP with synaptic density extended towards the thalamus, caudate nucleus and frontal regions. Plasma GFAP was also associated with GM volume, which partially (29%) mediated its relationship with synaptic density. However, a direct effect of synaptic density on plasma GFAP levels was also found.

Previous studies in CSF have consistently shown that SNAP25 levels are elevated in Alzheimer’s disease patients compared with healthy individuals as well as patients with other dementias like frontotemporal dementia and Lewy body dementia.^34,40,53,77-81^ Moreover, CSF SNAP25 levels are elevated in carriers of the *APOE-ɛ4* allele, the major genetic risk factor for Alzheimer’s disease, independently of clinical status, thus further supporting its relation to Alzheimer’s disease pathogenesis.^35,78,80^ A study in familial Alzheimer’s disease has shown that CSF SNAP25 levels start to increase as early as 15 years before estimated symptom onset, which closely follows increases of core CSF Alzheimer’s disease biomarkers like Aβ_1-42_/Aβ_1-40_ and pTau181 (17 and 18 years, respectively).^32^ In the current study, we did not find an association between SNAP25 levels in plasma and synaptic density in older adults without dementia. This apparent discrepancy might be a consequence of the relative specificity of SNAP25 for Alzheimer’s disease while Alzheimer’s disease pathology was largely absent in the current cohort (only 8% of participants that underwent amyloid PET demonstrated positivity). Of note, the specificity of SNAP25 for Alzheimer’s disease is not absolute as CSF SNAP25 elevations have also been observed in frontotemporal dementia, albeit to a lesser extent.^36,37,40^ Creutzfeldt-Jakob’s disease patients exhibit even higher CSF SNAP25 elevations than Alzheimer’s disease patients, likely due to its fulminant nature.^82,83^ Plasma levels of VAMP2, on the other hand, did demonstrate associations with synaptic density in temporal and frontal brain regions as well as within a small area within the occipital cortex. CSF VAMP2, like SNAP25, was previously shown to associate with CSF-based tau markers in Alzheimer’s disease and frontotemporal dementia patients as well as in individuals with no or subjective cognitive decline, indicating they are both linked to Alzheimer’s disease-related processes.^36,39,84^ However, unlike SNAP25, VAMP2 shows similar increases in the CSF of both Alzheimer’s disease and frontotemporal dementia patients relative to healthy controls, suggesting that its elevation is less dependent on Alzheimer’s disease pathology.^36,43^ In Lewy body dementia patients, CSF levels of both SNAP25 and VAMP2 are decreased compared with cognitively unimpaired controls, thus contrasting observations in both Alzheimer’s disease and frontotemporal dementia patients.^39^ It has been hypothesized that these decreases are a consequence of their intracellular sequestration induced by α-synuclein pathology. α-Synuclein directly binds to VAMP2, whose association with SNAP25 within the SNARE complex is essential for fusion pore formation enabling neurotransmitter release.^85^ Whether such SNAP25 and VAMP2 decreases also occur in plasma of Lewy body dementia patients remains to be determined. Of note, when Alzheimer’s disease co-pathology is present, Lewy body dementia patients demonstrate increased CSF levels of both SNARE proteins.^39,41^ Although increased CSF levels of synaptic proteins including SNAP25, VAMP2 and neurogranin have consistently been reported in Alzheimer’s disease, CSF-based SNAP25, VAMP2 and neurogranin decreases have been observed in the earliest phases of the clinical Alzheimer’s disease continuum.^31,38,79,84^ Moreover, for CSF VAMP2, fold changes (decreases) are largest in preclinical Alzheimer’s disease Stage 1 and are followed by gradual increases in later stages.^31,36,38^ CSF VAMP2 decreases have also been reported in individuals that demonstrate amyloid-positivity with normal levels of CSF pTau or neurodegeneration markers, regardless of their cognitive status.^31,38,86,87^ The biphasic profile of VAMP2 seen in clinical Alzheimer’s disease patients has led to the hypothesis that those with low CSF pTau181 levels might have different underlying pathological processes, resulting in decreased synaptic marker concentrations.^86^ However, CSF neurogranin, which increases with rising CSF tTau or pTau181 levels, is not lower in clinical Alzheimer’s disease patients with low CSF pTau levels than in healthy individuals.^86,88^ So far, decreases in neurogranin as well as SNAP25 in individuals without tau-mediated neurodegeneration have only been documented in preclinical Alzheimer’s disease stages.^31^ These early decreases in synaptic biomarkers, in either blood or CSF, might be due to early compensatory processes. Previous studies in Alzheimer’s disease mouse models showed enhanced synaptic activity in regions spared by Aβ pathology, but situated near regions of high Aβ burden, in order to compensate for the synaptic loss and to preserve brain function in early phases until pathology reaches a certain threshold.^3,4^ In the current study, VAMP2 did indeed associate with synaptic density outside of the typical predilection sites of pathology for the most common dementia types, i.e. Alzheimer’s disease (precuneus, cingulate gyrus, or orbitofrontal cortex)^89^ and frontotemporal dementia (basal ganglia, hippocampus).^90^ The compensatory role of decreases in synaptic markers is further supported by the earlier observation that decreased synaptic biomarkers, including VAMP2 and SNAP25, are associated with decreased, and thus more abnormal, CSF Aβ levels in cognitively unimpaired individuals^36^ or prodromal Alzheimer’s disease,^80^ whereas they increase with decreasing CSF Aβ levels in later clinical stages.^81,87^ Alternatively, interference of Aβ peptides in the interaction between SNAP25 and VAMP2 in early Alzheimer’s disease stages, similar to what is described for α-synuclein in Lewy body dementia, might explain the early decreases in CSF VAMP2 and SNAP25 levels.^39,91^ We did not observe an association between plasma VAMP2 and GM volume, thus suggesting its early involvement in pathogenesis. Plasma VAMP2 and SNAP25 correlated with each other, albeit moderately (*ρ* = 0.37 compared with 0.71–0.93 in CSF studies^31,36,39^). The contribution of peripheral sources to blood-based synaptic protein levels may, in part, explain the weaker correlation between VAMP2 and SNAP25 in blood compared with CSF.^92^ Such peripheral contribution appears especially relevant for VAMP2, for which we observed plasma concentrations ∼50 times higher than those of SNAP25, contrasting with the 4- to 10-fold difference that has been reported in CSF.^31,36,39^ Nonetheless, peripheral expression of SNAP25 has also been observed.^93^ Direct comparisons between CSF- and plasma-based levels of these synaptic proteins are required for a better insight into peripheral contributions. Moreover, blood is subjected to distinct degradation (e.g. proteases) and clearance (e.g. hepatic metabolism, renal excretion) pathways, which might decouple their concentrations, resulting in a weaker correlation. Plasma VAMP2 captured 28% of the total variance observed in SV2A PET, indicating a large portion of the variance remains unexplained. This further supports the likelihood of peripheral influences on plasma VAMP2 levels.

In addition to VAMP2, we also observed associations of GFAP with synaptic density, yet now within structures typically involved in dementia, such as the precuneus, cingulate gyrus and parahippocampus. In these regions, GM volume was also associated with GFAP and partially (29%) mediated its relationship with synaptic density. However, synaptic density also has a direct effect on plasma GFAP levels, independent of GM volume. This suggests that the observed association is not entirely driven by general neuronal loss, which is further supported by the absence of a correlation between plasma NfL, a marker of neurodegeneration, and synaptic density. Although blood-based GFAP increases, like SNAP25 increases, are more pronounced in Alzheimer’s disease compared with other neurodegenerative disorders such as frontotemporal dementia and Lewy body dementia,^94,95^ GFAP has demonstrated similar prognostic value for predicting both Alzheimer’s disease dementia and dementia due to other causes.^95-97^ In the current study population, Alzheimer’s disease pathology was largely absent, which makes it an unlikely driver of the observed relationships. To confirm this, we performed a sensitivity analysis in the subset with PET-based amyloid-negativity which revealed comparable associations of both GFAP and VAMP2 with synaptic density as those observed in the entire study population. The association between GFAP and GM volume was also comparable in this amyloid PET negative subset. GFAP, while not directly involved in the synaptic machinery, is intrinsically linked to synapses through its expression in astrocytes which play a crucial role in maintaining synaptic health. Astrocytic processes envelop synapses, forming the tripartite synapse, and regulate synaptic formation and elimination, thus contributing to the refinement of neuronal networks.^98,99^ Additionally, astrocytes provide structural support, regulate extracellular ion concentration and manage neurotransmitter uptake, all crucial for synaptic health.^99,100^ Increased GFAP expression and release has been found in several neurodegenerative disorders and indicate astrocyte reactivity in response to pathological environmental changes from early disease stages.^100^ Such reactivity results in aberrant synapse elimination which explains the observed negative association between GFAP and synaptic density.^101,102^ For the other biomarkers, pTau181, NfL, and Aβ_1-42_/Aβ_1-40_, no associations with synaptic density were observed. Moreover, none of the ATN(I) biomarkers associated with VAMP2 or SNAP25 in plasma. In contrast to previous reports by our own group^66,67^ and others,^103-105^ we did not observe a correlation between amyloid or tau PET load or *APOE-ɛ4* carrier status and any of the investigated biomarkers, including plasma Aβ_1-42_/Aβ_1-40_, pTau181, and GFAP, further suggesting that the results presented in this study were not driven by Alzheimer’s disease pathology and might apply to a wide variety of neurodegenerative disorders.^36,39,84^ Plasma pTau181 levels, however, did correlate with GFAP and NfL, which might reflect the presence of subthreshold Alzheimer’s disease neuropathology in a subset of participants that might not yet be detectable by PET. The lack of a correlation between plasma pTau181 and VAMP2 (or SNAP25) in the presence of subthreshold Alzheimer’s disease pathology in some participants may suggest that their associations differ between plasma and CSF, potentially reflecting differences in the timing of detectable changes in synaptic proteins versus pTau, or in their Alzheimer specificity across biofluids. Consistent with previous CSF studies, we found age-dependent increases in plasma levels of GFAP as well as NfL and pTau181,^70,72,104,106^ but not SNAP25, nor VAMP2.^42,78,82,107-109^ Studies that did find age-dependent changes in CSF SNAP25 or VAMP2 often included Alzheimer’s disease patients who were significantly older than controls, potentially confounding the results.^35,41,87^ Age-related changes in these synaptic proteins have, however, been reported in healthy individuals and preclinical Alzheimer’s disease, suggesting age might influence synaptic markers in those with no or low pathology.^31,39^ We could not replicate this result in our study as we found no age-dependency for either synaptic marker. Alternatively, when not including age as a covariate in voxelwise analyses, the observed associations of plasma VAMP2 with synaptic density were similar to those of age-adjusted models, yet now involving both hemispheres equally. The association of GFAP with synaptic density also overlapped with age-adjusted models, yet extended to the subcortical caudate nucleus, and thalamus, brain regions in which we, as well as earlier [^11^C]UCB-J PET studies in healthy older adults,^21,110^ found age-related synaptic loss. Moreover, in these brain regions, synaptic density was previously shown to be associated with the decline in movement quality and quantity that accompanies normal aging.^111^ Hence, in these individuals without dementia, plasma GFAP seems to reflect both disease-related and age-related synaptic loss.

This study has several limitations. First, the size of the study population is rather limited, which is an inherent consequence of the relative novelty of the [^11^C]UCB-J PET tracer. However, the sample size is comparable to, or even larger than, previous studies using this tracer.^19,112-114^ Another limitation is that our cohort included 23 (38%) participants with a diagnosis of late-life depression. Late-life depression is associated with reduced hippocampal volume and cortical thickness as well as increased neuroinflammation.^115^ However, we previously showed that [^11^C]UCB-J binding within the hippocampus and mesial temporal and prefrontal cortex is similar between patients with late-life depression and healthy controls, despite these patients demonstrating lower GM volume.^74^ This suggests that any potential synaptic density loss in late-life depression remains below the detection limit of our employed PET-based method and is unlikely to influence PET-based associations. We further limited the potential impact of depression on our results by correcting for late-life depression diagnosis in regression analyses as well as repeating the analysis after exclusion of patients with late-life depression. Importantly, since only late-life depression patients demonstrated cognitive impairment, this exclusion limited the analysis to cognitively unimpaired individuals. Moreover, only late-life depression patients received antidepressant treatment, which has been shown to affect synaptic density and brain VAMP2 levels, thereby also excluding potential antidepressant treatment effects in this sensitivity analysis.^116,117^ Whilst beyond the scope of the current study, further characterization of the effect of depression on plasma biomarkers could be informative. While mediation analysis suggested both a direct and indirect effect of synaptic density on plasma GFAP levels, the cross-sectional nature of the data limits causal inferences. Future longitudinal studies are needed to confirm the temporal sequence of these associations and to rule out unmeasured confounding (e.g. cerebrovascular disease, diet, physical activity, etc.). Moreover, since cross-sectional studies do not provide information on longitudinal trajectories of biomarkers or cognition, longitudinal studies assessing the prognostic value of blood-based synaptic biomarkers as well as their changes over time are needed to inform about their value for prognosis or monitoring in early disease stages. Amyloid and tau PET as well as plasma pTau181 data were not available for all included participants (respectively 12, 9, and 11 participants with missing data). Although this did not impede our primary objective to evaluate the association of plasma biomarkers with synaptic density, which were measured in all individuals (except for plasma pTau181), it did limit our ability to determine the influence of Alzheimer’s disease pathology of the observed associations. This ability was further limited by the overall low amyloid and tau burden among the included participants that did undergo amyloid and/or tau PET. Nevertheless, the absence of Alzheimer’s disease pathology enabled us to elucidate relationships between plasma biomarkers and synaptic density not specific to Alzheimer’s disease, which might thus be relevant for a wider variety of neurodegenerative diseases. Lastly, no CSF was available for L3D participants, which prevented comparisons of plasma SNAP25 and VAMP2 levels to their CSF counterparts, which are more established biomarkers of synapse pathology. Further validation of plasma SNAP25 and VAMP2 assays should include such comparisons.

## Conclusion

The current study demonstrates that elevated plasma levels of VAMP2 as well as GFAP correspond to lower synaptic density within distinct regions in the brains of older adults without dementia. Importantly, these associations were observed in absence of Alzheimer’s disease pathology and beyond GM loss, suggesting that plasma levels of VAMP2 and GFAP may reflect early synaptic alterations, not specific to Alzheimer’s disease and prior to substantial neurodegeneration. Such easily accessible blood-based biomarkers reflecting synaptic loss in various neurodegenerative disorders could aid in the early diagnosis as well as monitoring of treatment response in patients with neurodegenerative disorders.

## Supplementary Material

fcaf207_Supplementary_Data

## Data Availability

The data that support the findings of this study are available from the corresponding author, upon reasonable request. Codes generated and used within this work have been deposited at https://github.com/LAMONKUL/L3D_Synaptic.
