# Supplementary material for: Plasma vesicle-associated membrane protein 2 and glial fibrillary acidic protein associate with synaptic density in older adults without dementia
Source: Brain Commun. 2025 May 27;7(4):fcaf207. doi: 10.1093/braincomms/fcaf207 (PMC12225678; doi:10.1093/braincomms/fcaf207)
Supplement: fcaf207_Supplementary_Data [file fcaf207_supplementary_data.docx]

**SUPPLEMENTARY MATERIAL**

**Plasma vesicle-associated membrane protein 2 and glial fibrillary acidic protein associate with synaptic density in older adults without dementia**

Steffi De Meyer*, Soha Alali*, Maarten Laroy, Thomas Vande Casteele, Margot Van Cauwenberge, Julie Goossens, Charlotte De Rocker, Jeroen Vanbrabant, Eugeen Vanmechelen, Jan Van den Stock, Filip Bouckaert, Koen Van Laere, Mathieu Vandenbulcke^#^, Louise Emsell^#^, Koen Poesen^#^

*^*^Steffi De Meyer and Soha Alali contributed equally to this work.*

*^#^Mathieu Vandenbulcke, Louise Emsell and Koen Poesen contributed equally to this work.*

**Table of contents**

**Supplementary methods**

**Supplementary Tables**

- Supplementary Table 1. Plasma biomarker differences between late-life depression treatment groups
- Supplementary Table 2. Associations between plasma biomarkers and demographic variables
- Supplementary Table 3. Correlations between synaptic and ATN(I) plasma biomarkers
- Supplementary Table 4. Association of plasma biomarkers with grey matter volume in biomarker-specific VOIs
- Supplementary Table 5. Demographics of the study population stratified by amyloid PET status
- Supplementary Table 6. Sensitivity analyses in amyloid PET-negative older adults without dementia
- Supplementary Table 7. Demographics of the study population stratified by late-life depression diagnosis
- Supplementary Table 8. Sensitivity analyses in cognitively unimpaired older adults without late-life depression diagnosis

**Supplementary Figures**

- Supplementary Fig. 1. Distribution of brain amyloid and tau PET load within the L3D cohort
- Supplementary Fig. 2. The association of plasma NfL levels with synaptic density, adjusted for age, sex, and late-life depression diagnosis
- Supplementary Fig. 3. The association of plasma VAMP2 and GFAP levels with synaptic density, adjusted for sex and late-life depression diagnosis
- Supplementary Fig. 4. The association of age with synaptic density
- Supplementary Fig. 5. Mediation analyses in study population subsets

**Supplementary methods.**

An ADx in-house VAMP2 research prototype assay on the Simoa platform was developed with homebrew assay development kit components from Quanterix (#101354, Quanterix, Billerica, USA). 0.25 mg/mL of mouse monoclonal RD-087 antibody was coupled to 2E+08 paramagnetic Quanterix carboxyl beads. The analyte VAMP2 was detected in plasma samples by using 2E+07 active RD-087 coupled capture beads/mL (50% helper beads) and 0.5 µg/mL of mouse RD-081 detector antibody diluted in a well-optimized in-house sample diluent. A 60-minute incubation (80 cadences) in a cuvette was performed with 25 µL of the RD-087 coupled beads solution, 1:3 diluted plasma sample, and 20 µL of the RD-081 detector antibody solution. After a wash step, a second incubation of 5 minutes and 15 seconds (7 cadences) was conducted where 100 µL of 50 pM streptavidin-β-galactosidase (SBG) solution was added to the cuvette, followed by another wash step. Lastly, resorufin β-D-galactopyranoside (RGP) substrate solution was added to the solution and loaded on to the Simoa disc array, resulting in fluorescent signals from which the VAMP2 concentrations could be extrapolated using a well-defined calibrator curve based on a peptide (aa1-69).

An ADx in-house SNAP25 research prototype assay on the Simoa platform was developed with homebrew assay development kit components from Quanterix (#101354, Quanterix, Billerica, USA). 0.3 mg/mL of mouse monoclonal ADx404 antibody was coupled to 1.34E+08 Agilent Lodestars High Bind carboxyl beads (PL6827-0003, Santa Clara, California, USA) and incubated together with the monoclonal mouse RD-086 detector antibody. The analyte SNAP25 was detected in plasma samples by using 1.50E+07 active ADx404 coupled capture beads/mL (50% helper beads) and 0.5 µg/mL of RD-086 detector antibody diluted in a PBS/casein based homebrew diluent. A 60-minute incubation (80 cadences) in a cuvette was performed with 25 µL of the ADx404 coupled beads solution, 1:8 diluted plasma sample, and 20 µL of the RD-086 detector antibody solution. After a wash step, a second incubation of 5 minutes and 15 seconds (7 cadences) was conducted where 100 µL of 50 pM SBG solution was added to the cuvette, followed by another wash step. Lastly, RGP substrate solution was added to the solution and loaded on to the Simoa disc array, resulting in fluorescent signals from which the SNAP25 concentrations could be extrapolated using a well-defined calibrator curve based on an acetylated peptide (aa2-40).

The analytical lower limit of quantification was 0.05 pg/mL for SNAP25 and 2 pg/mL for VAMP2. Parallelism, spike recovery and dilution linearity were within the acceptable range of 80% to 120%. Repeatability and reproducibility in plasma were, respectively, 2.3 to 4.5 CV% and 5.4 to 7.9 CV% for SNAP25 and 4.5 to 7.9 CV% and 14 to 19 CV% for VAMP2.

**Supplementary Table 1**. Plasma biomarker differences between late-life depression treatment groups

| **Drug** | **Subgroup** | **VAMP2** | **SNAP25** | **GFAP** | **NfL** | **Aβ_1-42_/Aβ_1-40_** | **pTau181** |
| --- | --- | --- | --- | --- | --- | --- | --- |
| anticholinergics | treated (*n* = 3) | 29.8 | 0.76 | 149 | 16.4 | 0.065 | 13.3 |
|  | untreated (*n* = 20) | 35.3 | 0.84 | 123 | 24.6 | 0.065 | 18.1 |
|  | Fold increase | 0.84 | 0.91 | 1.21 | 0.67 | 1.00 | 0.74 |
|  | *P_uncorrected_* | 0.97 | 0.23 | 0.17 | 0.83 | 0.70 | 0.44 |
|  | *P_Bonferroni_* | > 0.99 | > 0.99 | > 0.99 | > 0.99 | > 0.99 | > 0.99 |
| antipsychotics | treated (*n* = 14) | 32.2 | 0.78 | 123 | 22.6 | 0.064 | 16.4 |
|  | untreated (*n* = 9) | 42.2 | 0.89 | 133 | 27.8 | 0.070 | 19.7 |
|  | Fold increase | 0.76 | 0.88 | 0.93 | 0.81 | 0.92 | 0.83 |
|  | *P_uncorrected_* | 0.83 | 0.34 | 0.64 | 0.78 | 0.69 | 0.76 |
|  | *P_Bonferroni_* | > 0.99 | > 0.99 | > 0.99 | > 0.99 | > 0.99 | > 0.99 |
| antidepressants | treated (*n* = 22) | 35.3 | 0.80 | 130 | 24.6 | 0.064 | 17.0 |
|  | untreated (*n* = 1) | 18.7 | 0.93 | 97 | 14.3 | 0.082 | 14.0 |
|  | Fold increase | 1.89 | 0.86 | 1.34 | 1.73 | 0.79 | 1.21 |
|  | *P_uncorrected_* | 0.087 | 0.52 | 0.78 | 0.26 | 0.43 | 0.80 |
|  | *P_Bonferroni_* | 0.52 | > 0.99 | > 0.99 | > 0.99 | > 0.99 | > 0.99 |
| benzodiazepines | treated (*n* = 11) | 32.0 | 0.76 | 124 | 22.0 | 0.062 | 15.5 |
|  | untreated (*n* = 12) | 35.3 | 0.82 | 130 | 27.4 | 0.072 | 18.1 |
|  | Fold increase | 0.91 | 0.93 | 0.96 | 0.80 | 0.87 | 0.86 |
|  | *P_uncorrected_* | 0.88 | 0.69 | 0.98 | 0.26 | 0.38 | 0.85 |
|  | *P_Bonferroni_* | > 0.99 | > 0.99 | > 0.99 | > 0.99 | > 0.99 | > 0.99 |
| opiates | treated (*n* = 2) | 36.6 | 0.84 | 133 | 41.5 | 0.073 | 28.9 |
|  | untreated (*n* = 21) | 35.3 | 0.79 | 124 | 22.7 | 0.064 | 15.9 |
|  | Fold increase | 1.04 | 1.06 | 1.08 | 1.83 | 1.14 | 1.83 |
|  | *P_uncorrected_* | 0.96 | 0.79 | 0.71 | 0.24 | 0.57 | 0.32 |
|  | *P_Bonferroni_* | > 0.99 | > 0.99 | > 0.99 | > 0.99 | > 0.99 | > 0.99 |

Concentrations of the measured biomarkers are presented in pg/mL for late-life depression patients treated and untreated with, respectively, anticholinergics, antipsychotics, antidepressants, benzodiazepines and opiates. Fold increases are defined as the biomarker concentration in the treated late-life depression subgroup divided by the biomarker concentration in the untreated subgroup. Group comparisons were performed using Mann-Whitney U tests. *P* values are reported both uncorrected (*P_uncorrected_*) and Bonferroni-corrected (*P_Bonferroni_*) for multiple comparisons (*k* = 6 biomarkers).

**Supplementary Table 2.** Associations between plasma biomarkers and demographic variables

| **Variable** | **VAMP2** | **SNAP25** | **GFAP** | **NfL** | **Aβ_1-42_/Aβ_1-40_** | **pTau181** |
| --- | --- | --- | --- | --- | --- | --- |
| Sex |  |  |  |  |  |  |
| W | 479 | 347 | 361 | 370 | 438 | 214 |
| *P_uncorrected_* | 0.46 | 0.22 | 0.31 | 0.38 | 0.90 | 0.090 |
| *P_Bonferroni_* | > 0.99 | > 0.99 | > 0.99 | > 0.99 | > 0.99 | 0.54 |
| APOE |  |  |  |  |  |  |
| W | 203 | 188 | 263 | 266 | 352 | 182.5 |
| *P_uncorrected_* | 0.32 | 0.20 | 0.88 | 0.84 | 0.060 | 0.97 |
| *P_Bonferroni_* | > 0.99 | > 0.99 | > 0.99 | > 0.99 | 0.36 | > 0.99 |
| Education |  |  |  |  |  |  |
| χ^2^ | 1.30 | 3.16 | 3.02 | 1.50 | 0.90 | 0.91 |
| *P_uncorrected_* | 0.73 | 0.37 | 0.39 | 0.68 | 0.83 | 0.82 |
| *P_Bonferroni_* | > 0.99 | > 0.99 | > 0.99 | > 0.99 | > 0.99 | > 0.99 |
| Age |  |  |  |  |  |  |
| ρ | -0.09 | -0.01 | 0.52 | 0.53 | 0.21 | 0.53 |
| 95% CI | -0.33 to 0.15 | -0.27 to 0.25 | 0.31 to 0.70 | 0.29 to 0.71 | -0.02 to 0.42 | 0.28 to 0.72 |
| *P_uncorrected_* | 0.50 | 0.93 | < 0.0001 | < 0.0001 | 0.11 | < 0.0001 |
| *P_Bonferroni_* | > 0.99 | > 0.99 | < 0.0001 | < 0.0001 | 0.66 | 0.00048 |
| Amyloid PET |  |  |  |  |  |  |
| ρ | -0.19 | -0.04 | 0.07 | 0.17 | -0.08 | -0.02 |
| 95% CI | -0.44 to 0.11 | -0.31 to 0.24 | -0.26 to 0.39 | -0.12 to 0.47 | -0.35 to 0.20 | -0.35 to 0.33 |
| *P_uncorrected_* | 0.20 | 0.79 | 0.62 | 0.24 | 0.56 | 0.89 |
| *P_Bonferroni_* | > 0.99 | > 0.99 | > 0.99 | > 0.99 | > 0.99 | > 0.99 |
| Tau PET |  |  |  |  |  |  |
| ρ | 0.04 | 0.18 | -0.07 | 0.06 | 0.17 | 0.21 |
| 95% CI | -0.24 to 0.30 | -0.09 to 0.41 | -0.37 to 0.22 | -0.22 to 0.33 | -0.10 to 0.42 | -0.13 to 0.50 |
| *P_uncorrected_* | 0.76 | 0.20 | 0.62 | 0.65 | 0.23 | 0.18 |
| *P_Bonferroni_* | > 0.99 | > 0.99 | > 0.99 | > 0.99 | > 0.99 | > 0.99 |

The relationships of plasma biomarkers with demographic variables or other biomarker modalities were calculated using Mann-Whitney U tests for binary variables, Kruskal-Wallis tests for categorical variables and Spearman correlations for continuous variables. Test statistics and corresponding *P* values are shown both uncorrected (*P_uncorrected_*) and corrected (*P_Bonferroni_*) for multiple comparisons (*k* = 6 biomarkers).

**Supplementary Table 3.** Correlations between synaptic and ATN(I) plasma biomarkers

| **Variable** | **Statistic** | **SNAP25** | **GFAP** | **NfL** | **Aβ_1-42_/Aβ_1-40_** | **pTau181** |
| --- | --- | --- | --- | --- | --- | --- |
| VAMP2 | ρ | 0.37 | -0.01 | -0.22 | -0.17 | -0.24 |
|  | 95% CI | 0.14 to 0.57 | -0.26 to 0.22 | -0.46 to 0.05 | -0.42 to 0.09 | -0.44 to 0.006 |
|  | *P_uncorrected_* | 0.0032 | 0.92 | 0.095 | 0.19 | 0.095 |
|  | *P_Bonferroni_* | 0.019 | > 0.99 | 0.57 | > 0.99 | 0.57 |
| SNAP25 | ρ | 1 | 0.09 | 0.04 | 0.06 | -0.13 |
|  | 95% CI |  | -0.20 to 0.36 | -0.19 to 0.28 | -0.24 to 0.34 | -0.38 to 0.15 |
|  | *P_uncorrected_* |  | 0.50 | 0.74 | 0.66 | 0.38 |
|  | *P_Bonferroni_* |  | > 0.99 | > 0.99 | > 0.99 | > 0.99 |
| GFAP | ρ |  | 1 | 0.58 | 0.12 | 0.55 |
|  | 95% CI |  |  | 0.38 to 0.74 | -0.14 to 0.34 | 0.30 to 0.74 |
|  | *P_uncorrected_* |  |  | < 0.0001 | 0.34 | < 0.0001 |
|  | *P_Bonferroni_* |  |  | < 0.0001 | > 0.99 | < 0.0001 |
| NfL | ρ |  |  | 1 | 0.31 | 0.57 |
|  | 95% CI |  |  |  | 0.07 to 0.51 | 0.32 to 0.74 |
|  | *P_uncorrected_* |  |  |  | 0.016 | < 0.0001 |
|  | *P_Bonferroni_* |  |  |  | 0.096 | < 0.0001 |
| Aβ_1-42_/Aβ_1-40_ | ρ |  |  |  | 1 | 0.21 |
|  | 95% CI |  |  |  |  | -0.06 to 0.47 |
|  | *P_uncorrected_* |  |  |  |  | 0.15 |
|  | *P_Bonferroni_* |  |  |  |  | 0.90 |

Spearman correlation coefficients with corresponding *P* values and 95% confidence intervals (CI, bootstrapping, *n* = 1,000) are shown for the correlation between all measured plasma biomarkers across study participants. *P* values are reported both uncorrected (*P_uncorrected_*) and corrected (*P_Bonferroni_*) for multiple comparisons (*k* = 6 biomarkers).

**Supplementary Table 4.** Association of plasma biomarkers with grey matter volume in biomarker-specific VOIs

| Outcome | *R^2^* | *F* | Predictor | β_s_ [95% CI] | *P_uncorrected_* | *P_Bonferroni_* |
| --- | --- | --- | --- | --- | --- | --- |
| ln(VAMP2) | 0.03 | F(3,57) = 0.61 | GM | -0.11 [-0.33 to 0.11] | 0.31 | 0.62 |
|  |  |  | Age | -0.01 [-0.06 to 0.03] | 0.54 | > 0.99 |
|  |  |  | Sex | -0.19 [-0.70 to 0.32] | 0.47 | 0.93 |
| GFAP | 0.38 | F(3,57) = 11.81 | GM | -0.15 [-0.25 to -0.05] | 0.0038 | 0.0077 |
|  |  |  | Age | 0.05 [0.01 to 0.09] | 0.017 | 0.034 |
|  |  |  | Sex | 0.45 [0.01 to 0.89] | 0.043 | 0.086 |

*R^2^* values, *F* statistics and and standardied effect estimates (βs) with corresponding 95% CIs were derived from multiple regression models constructed in the entire Leuven late-life depression (L3D) cohort (*n* = 61). Grey matter (GM) volumes were calculated in the respective biomarker-specific volumes of interest (VOIs). Both models were corrected for age and sex. To meet model assumptions, vesicle-associated membrane protein 2 (VAMP2) concentrations were natural log (ln) transformed. Glial fibrillary acidic protein (GFAP) levels were not transformed. Biomarker values were scaled (*z*-scored) to enable direct interbiomarker comparison of effect sizes. *P* values are reported both uncorrected (*P_uncorrected_*) and corrected (*P_Bonferroni_*) for multiple comparisons (*k* = 2 biomarkers).

**Supplementary Table 5.** Demographics of the study population stratified by amyloid PET status

| **Characteristics** | ***n*** | **A-** | ***n*** | **A+** | ***n*** | **unknown** |
| --- | --- | --- | --- | --- | --- | --- |
| Female, no. (%) | 45 | 29 (64) | 4 | 2 (50) | 12 | 8 (67) |
| Age, years | 45 | 71 ± 6 | 4 | 74 [5] | 12 | 72 [8] |
| Education level (1/2/3/4)^a^, no. (%) | 45 | 6/19/14/6 | 4 | 1/1/2/0 | 12 | 0/10/2/0 |
| MMSE, /30 | 45 | 29 [2] | 4 | 26 [3] | 12 | 28 [6] |
| *APOE-ε4* carriers, no. (%) | 45 | 5 (11) | 4 | 3 (75) | 12 | 2 (17) |
| Depression diagnosis, no. (%) | 45 | 11 (24) | 4 | 1 (25) | 12 | 11 (92) |
| Aβ load, SUVR | 45 | 1.19 [0.07] | 4 | 1.82 [0.11] | 0 | *NA* |
| Tau load, SUVR | 45 | 0.97 ± 0.15 | 4 | 0.89 [0.06] | 3 | 0.77 [0.20] |
| SV2A PET, SUVR_comp,VAMP2_ | 45 | 3.70 ± 1.28 | 4 | 4.28 [1.32] | 3 | 3.99 [2.26] |
| SV2A PET, SUVR_comp,GFAP_ | 45 | 5.32 ± 0.75 | 4 | 5.41 [1.50] | 3 | 5.13 [0.66] |
| Plasma Aβ_1-42_/Aβ_1-40_ | 45 | 0.066 ± 0.012 | 4 | 0.054 [0.004] | 12 | 0.065 [0.025] |
| Plasma pTau181, pg/mL | 37 | 12.6 [14.6] | 3 | 14.5 [15.1] | 10 | 15.9 [11.4] |
| Plasma GFAP, pg/mL | 45 | 89 [68] | 4 | 133 [16] | 12 | 137 [85] |
| Plasma NfL, pg/mL | 45 | 17.4 [10.3] | 4 | 14.7 [9.0] | 12 | 25.4 [18.1] |
| Plasma VAMP2, pg/mL | 45 | 49 [33] | 4 | 67 [46] | 12 | 39 [14] |
| Plasma SNAP25, pg/mL | 45 | 0.94 [0.33] | 4 | 0.93 [0.05] | 12 | 0.83 [0.19] |

For the amyloid PET-negative (A-) subgroup, continuous data are expressed as mean ± standard deviation (SD) when normally distributed and median [interquartile range (IQR)] when not. Categorical data are expressed as number (%). Due to the small sample size of the amyloid PET-negative subgroup (A-) no statistical comparison of demographic/biomarker variables was performed between subgroups, and for the A+ subgroup and the subgroup with unknown amyloid status, continuous data are expressed as median [IQR]. *NA*, not applicable.

^a^The highest attained levels of education were defined as follows: 1 = primary education, 2 = secondary education, 3 = higher education, 4 = university

**Supplementary Table 6.** Sensitivity analyses in amyloid PET-negative older adults without dementia

| Outcome | *R^2^* | *F* | Predictor | β_s_ [95% CI] | *P_uncorrected_* | *P_Bonferroni_* |
| --- | --- | --- | --- | --- | --- | --- |
| SV2A | 0.31 | F(1, 43) = 19.05 | VAMP2 | -0.71 [-1.03 to -0.38] | < 0.0001 | 0.00016 |
| SV2A | 0.22 | F(1,43) = 11.79 | GFAP | -0.35 [-0.55 to -0.14] | 0.0013 | 0.0027 |
| ln(VAMP2) | 0.02 | F(3,41) = 0.22 | GM | -0.10 [-0.36 to 0.17] | 0.46 | 0.93 |
|  |  |  | Age | -0.01 [-0.07 to 0.06] | 0.84 | > 0.99 |
|  |  |  | Sex | -0.001 [-0.66 to 0.66] | 0.99 | > 0.99 |
| GFAP | 0.33 | F(3,41) = 6.75 | GM | -0.16 [-0.27 to -0.04] | 0.010 | 0.020 |
|  |  |  | Age | 0.03 [-0.02 to 0.08] | 0.29 | 0.58 |
|  |  |  | Sex | 0.56 [0.02 to 1.10] | 0.044 | 0.087 |

*R^2^* values, *F* statistics and and standardized effect estimates (β_s_) with corresponding 95% confidence intervals (CIs) were derived from multiple regression models constructed in the subset of the study population that were amyloid-PET negative (*n* = 45). For each biomarker, grey matter (GM) volume and composite synaptic vesicle glycoprotein 2A (SV2A) standardized uptake value ratios (SUVRs) were calculated in the respective biomarker-specific volumes of interest (VOIs). For each model, the predictor and outcome variables are shown in the table. To meet model assumptions, vesicle-associated membrane protein 2 (VAMP2) concentration were natural log (ln) transformed in regression models evaluating the association with grey matter volume. Glial fibrillary acidic protein (GFAP) levels were not transformed. Biomarker values were scaled (*z*-scored) to enable interbiomarker comparison of effect sizes. *P* values are reported both uncorrected (*P_uncorrected_*) and corrected (*P_Bonferroni_*) for multiple comparison (*k* = 2 biomarkers).

**Supplementary Table 7.** Demographics of the study population stratified by late-life depression diagnosis

| **Characteristics** | ***n*** | **Healthy** | ***n*** | **Late-life depression** | ***P*** |
| --- | --- | --- | --- | --- | --- |
| Female, no. (%) | 38 | 24 (63) | 23 | 15 (65) | > 0.99 |
| Age, years | 38 | 71 ± 6 | 23 | 74 ± 6 | 0.17 |
| Education level (1/2/3/4)^a^, no. (%) | 38 | 3/15/15/5 | 23 | 4/15/3/1 | 0.056 |
| MMSE, /30 | 38 | 29 [2] | 23 | 27 [5] | < 0.0001 |
| *APOE-ε4* carriers, no. (%) | 38 | 6 (16) | 23 | 4 (17) | > 0.99 |
| Aβ load, SUVR | 37 | 1.19 [0.06] | 12 | 1.24 [0.96] | 0.23 |
| Aβ positive, no. (%) | 38 | 3 (8) | 12 | 1 (8) | > 0.99 |
| Tau load, SUVR | 38 | 0.97 ± 0.15 | 14 | 0.81 ± 0.15 | 0.42 |
| SV2A PET, SUVR_comp,VAMP2_ | 38 | 3.62 ± 1.26 | 23 | 4.04 ± 1.36 | 0.24 |
| SV2A PET, SUVR_comp,GFAP_ | 38 | 5.29 ± 0.74 | 23 | 5.37 ± 0.81 | 0.72 |
| Plasma Aβ_1-42_/Aβ_1-40_ | 38 | 0.064 ± 0.013 | 23 | 0.068 ± 0.013 | 0.23 |
| Plasma pTau181, pg/mL | 30 | 10.5 [14.4] | 20 | 16.7 [14.2] | 0.054 |
| Plasma GFAP, pg/mL | 38 | 100 ± 45 | 23 | 121 ± 46 | 0.60 |
| Plasma NfL, pg/mL | 38 | 14.4 [9.1] | 23 | 24.3 [14.4] | 0.00072 |
| Plasma VAMP2, pg/mL | 38 | 58 [29] | 23 | 35 [15] | < 0.0001 |
| Plasma SNAP25, pg/mL | 38 | 0.99 ± 0.24 | 23 | 0.82 ± 0.16 | 0.012 |

Continuous data are expressed as mean ± standard deviation (SD) when normally distributed and median [interquartile range (IQR)] when not. Categorical data are expressed as number (%). Reported *P* values for biomarker differences were Bonferroni-corrected for multiple comparisons (*k* = 6 biomarkers)

^a^The highest attained levels of education were defined as follows: 1 = primary education, 2 = secondary education, 3 = higher education, 4 = university

**Supplementary Table 8.** Sensitivity analyses in cognitively unimpaired older adults without late-life depression diagnosis

| Outcome | *R^2^* | *F* | Predictor | β_s_ [95% CI] | *P_uncorrected_* | *P_Bonferroni_* |
| --- | --- | --- | --- | --- | --- | --- |
| SV2A ~ VAMP2 | 0.35 | F (1,36) = 19.80 | VAMP2 | -0.75 [-1.09 to -0.41] | < 0.0001 | 0.00016 |
| SV2A ~ GFAP | 0.21 | F (1,36) = 9.53 | GFAP | -0.35 [-0.57 to -0.12] | 0.0039 | 0.0078 |
| ln(VAMP2) | 0.07 | F(3,34) = 0.86 | GM | -0.11 [-0.38 to 0.16] | 0.40 | 0.80 |
|  |  |  | Age | -0.001 [-0.06 to 0.06] | 0.98 | > 0.99 |
|  |  |  | Sex | -0.33 [-1.01 to 0.35] | 0.33 | 0.67 |
| GFAP | 0.28 | F(3,34) = 4.47 | GM | -0.14 [-0.26 to -0.01] | 0.030 | 0.060 |
|  |  |  | Age | 0.03 [-0.02 to 0.09] | 0.27 | 0.54 |
|  |  |  | Sex | 0.37 [-0.23 to 0.98] | 0.22 | 0.44 |

*R^2^* values, *F* statistics and and standardized effect estimates (β_s_) with corresponding 95% CIs were derived from multiple regression models in the subset of the study population that were not diagnosed with late-life depression (*n* = 38). For each biomarker, grey matter (GM) volume and composite synaptic vesicle glycoprotein 2A (SV2A) standardized uptake value ratios (SUVRs) were calculated in the respective biomarker-specific volumes of interest (VOIs). For each model, the predictor and outcome variables are shown in the table. that participants without late-life depression diagnosis were also cognitively unimpaired whereas participants with late-life depression diagnosis demonstrated some degree of cognitive impairment. To meet model assumptions, vesicle-associated membrane protein 2 (VAMP2) concentrations were natural log (ln) transformed in regression models evaluating the association with grey matter volume. Glial fibrillary acidic protein (GFAP) levels were not transformed. Biomarker values were scaled (*z*-scored) to enable direct interbiomarker comparison of effect sizes. *P* values are reported both uncorrected (*P_uncorrected_*) and corrected (*P_Bonferroni_*) for multiple comparisons (*k* = 2 biomarkers).


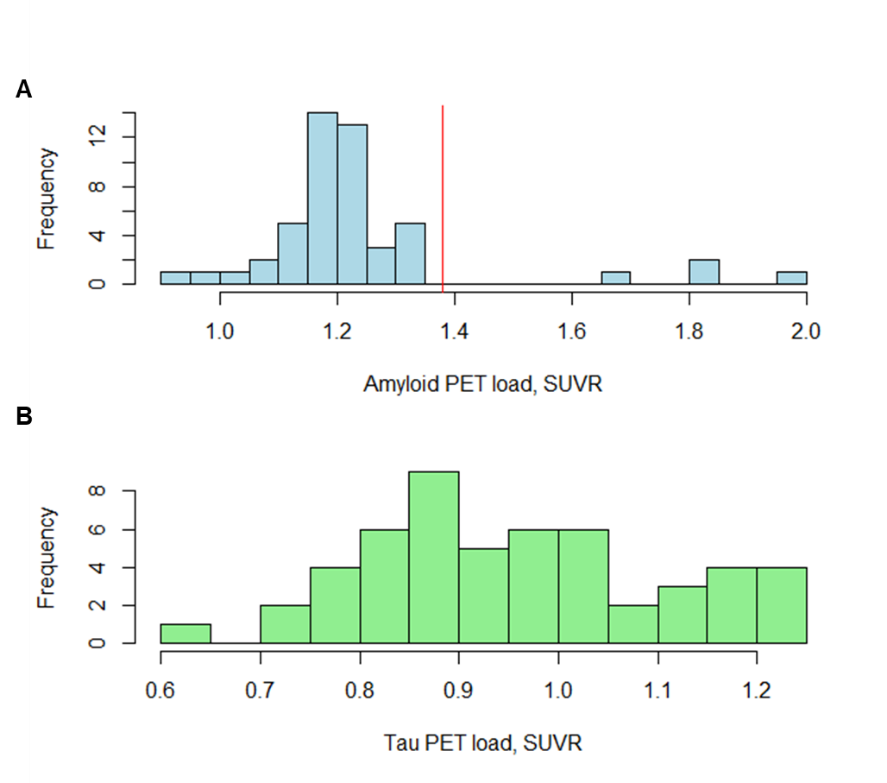
**Supplementary Fig. 1.** Distribution of brain amyloid and tau PET load within the L3D cohort

Histograms of amyloid ([^18^F]flutemetamol, **A**, blue, *n* = 49) and tau ([^18^F]MK6240 tau, **B**, green, *n* = 52) PET standardized uptake value ratios (SUVRs) are shown. The vertical red line represents the threshold of amyloid PET positivity (SUVR > 1.38).

**Supplementary Fig. 2.** The association of plasma NfL levels with synaptic density, adjusted for age, sex, and late-life depression diagnosis


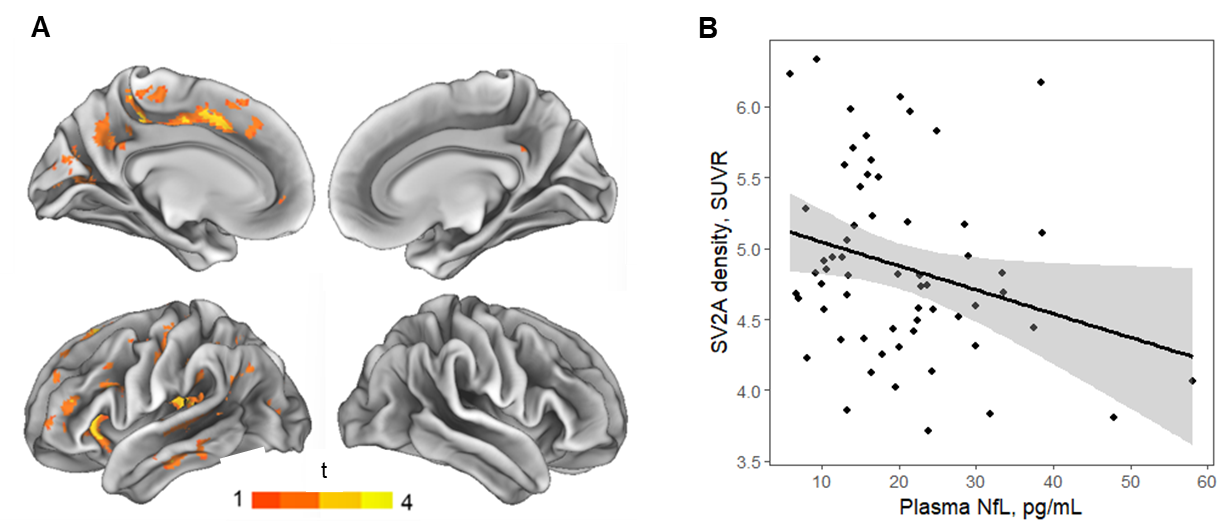


**A**) Parametric *T* maps of the regional associations between plasma neurofilament light (NfL) levels and synaptic vesicle glycoprotein 2A (SV2A) PET standardized uptake value ratios (SUVRs) in the Leuven late-life depression (L3D) cohort (*n* = 61), which were calculated through voxelwise multiple linear regression models adjusted for age, sex and a diagnosis of late-life depression. The significance threshold was set at a cluster-level whole-brain family-wise error (FWE) threshold of *P_FWE_* < 0.05 with voxel-level set at *P_uncorrected_* < 0.05. Thresholded maps were superimposed on the left and right hemisphere of the PALS cortical surface (PALS-B12) using CARET v5.65.^1^ **B**) Scatterplot of plasma NfL and composite SV2A PET SUVR in the identified biomarker-specific volume of interest (VOI), in which the association strength was quantified by means of the standardized regression coefficient from simple linear models (NfL as predictor, composite SV2A PET SUVR as outcome). Each data point represents an L3D study participant (*n* = 61). Linear fits (black lines) with corresponding 95% CIs (shaded area) as derived from simple linear regression models using plasma NfL as predictor and composite SV2A PET SUVR as outcome are superimposed.

1. Van Essen DC, Drury HA, Dickson J, Harwell J, Hanlon D, Anderson CH. An integrated software suite for surface-based analyses of cerebral cortex. J Am Med Inform Assoc. 2001;8:443–59.


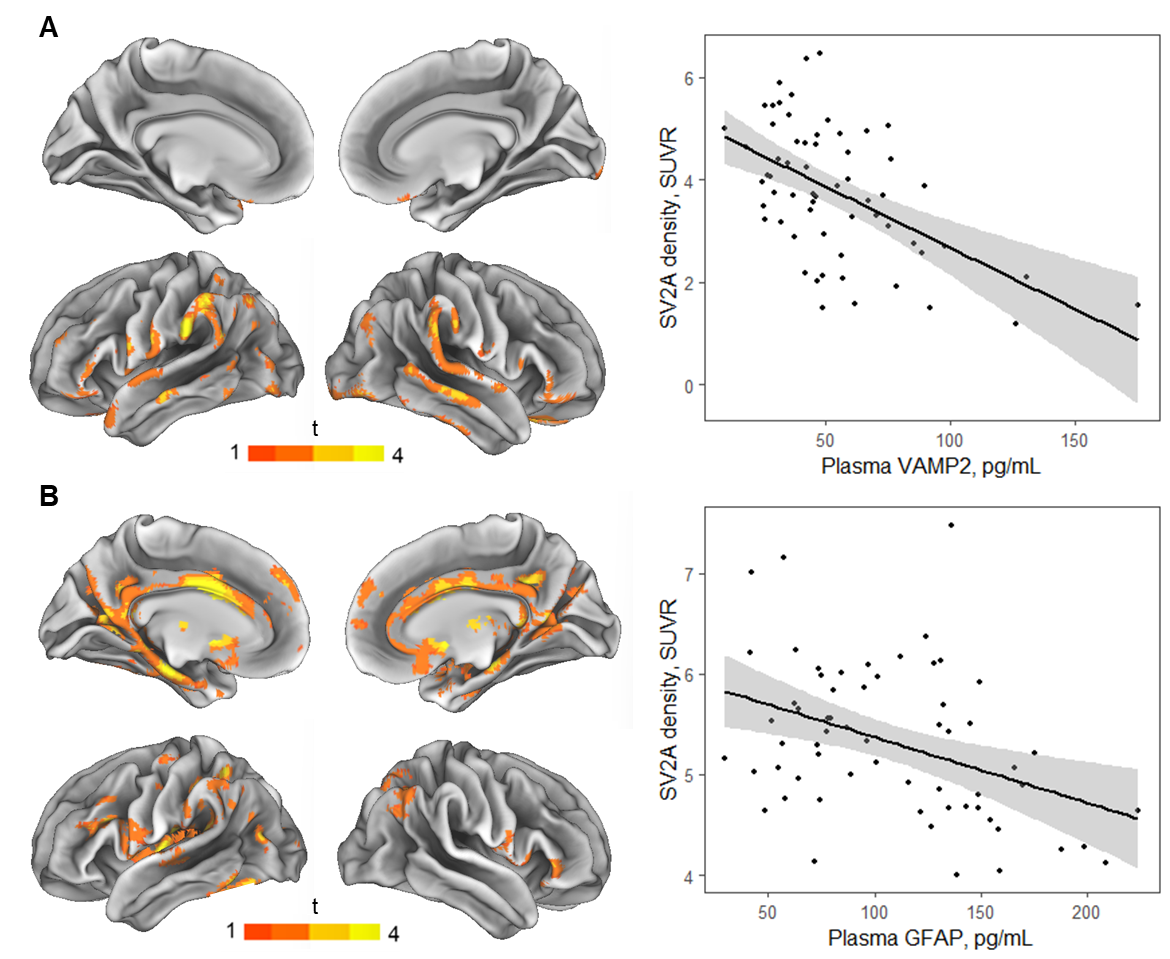
**Supplementary Fig. 3.** The association of plasma VAMP2 and GFAP levels with synaptic density, adjusted for sex and late-life depression diagnosis

The left panels show the parametric *T* maps of the regional associations of plasma vesicle-associated membrane protein 2 (VAMP2, **A**, top) and glial fibrillary acidic protein (GFAP, **B**, bottom) levels with synaptic vesicle glycoprotein 2A (SV2A) PET standardized uptake values ratio (SUVR) in the Leuven late-life depression (L3D) cohort (*n* = 61), which were calculated through voxelwise multiple linear regression models adjusted for sex and depression diagnosis, but not for age. The significance threshold was set at a cluster-level whole-brain family-wise error (FWE) threshold of *P_FWE_* < 0.05 with voxel-level set at *P_uncorrected_* < 0.05. Thresholded maps were superimposed on the left and right hemisphere of the PALS cortical surface (PALS-B12) using CARET v5.65.^1^ The right panels show the scatterplot of plasma VAMP2 and GFAP, respectively, with the composite SV2A PET SUVR_comp_ in the respective identified volumes of interest (VOIs). Clusters showing significant associations (shown in the left panels) were used as biomarker-specific VOIs, in which the association strength was quantified by means of the standardized regression coefficient from simple linear models (biomarker as predictor, composite SV2A PET SUVR as outcome). Each data point represents an L3D study participant (*n* = 61). Linear fits (black lines) with corresponding 95% confidence intervals (CIs, shaded area) as derived from simple linear regression models using plasma VAMP2 (**A**) or GFAP (**B**) as predictor and SV2A PET as outcome are superimposed. Note that raw biomarker values were plotted to ease interpretation, but statistical analyses were performed on scaled biomarker values (*z*-scores) to enable direct comparison of effect sizes.

1. Van Essen DC, Drury HA, Dickson J, Harwell J, Hanlon D, Anderson CH. An integrated software suite for surface-based analyses of cerebral cortex. J Am Med Inform Assoc. 2001;8:443–59.

**Supplementary Fig. 4.** The association of age with synaptic density


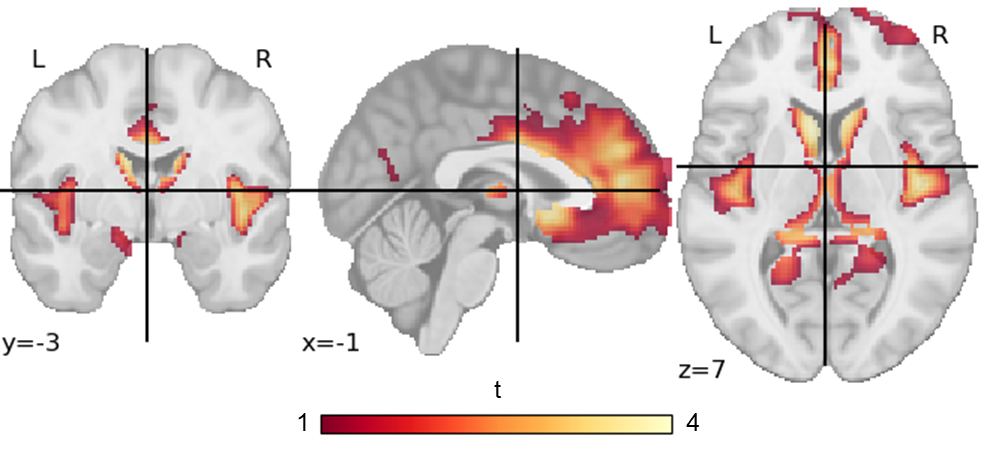


Parametric *T* maps show the association of age (predictor) with synaptic vesicle glycoprotein 2A (SV2A) PET standardized uptake value ratios (SUVRs, outcome) as calculated through voxelwise simple regression models with age as predictor and SV2A PET SUVR images as outcome in the Leuven late-life depression (L3D) cohort (*n* = 61). Thresholded maps (voxel-level *P_uncorrected_* < 0.05, cluster level *P_FWE_* < 0.05) were superimposed on the MNI152 template using the Nilearn package in Python (v3.9.13). Crosshairs indicate the cut positions of the coronal, sagittal and transverse planes.


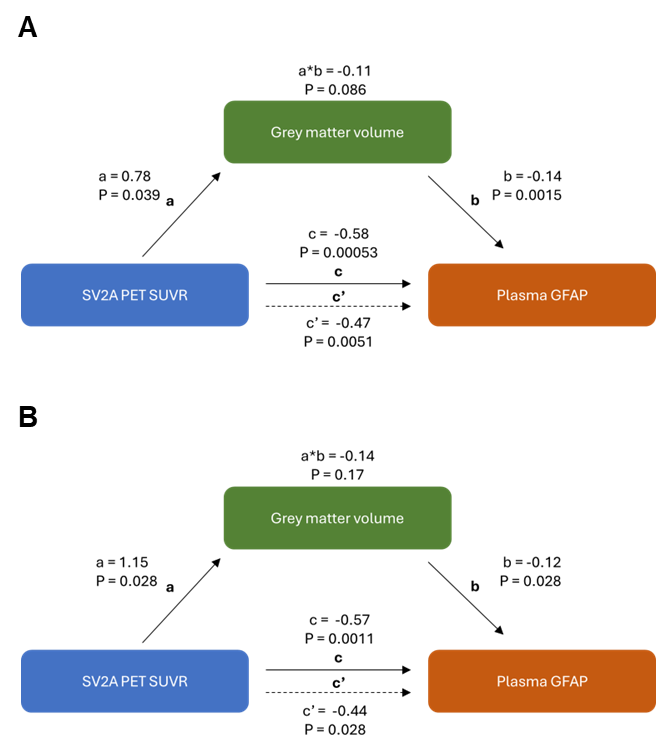
**Supplementary Fig. 5.** Mediation analyses in study population subsets.

The mediation models contain composite synaptic vesicle glycoprotein 2A (SV2A) PET standardized uptake value ratios (SUVRs) in the biomarker-specific volume of interest (as measured by [^11^C]UCB-J PET) as a predictor, grey matter volume in the biomarker-specific volume of interest as a mediator, and plasma glial fibrillary acidic protein (GFAP) as outcome in the Leuven late-life depression (L3D) subsets that were amyloid-negative (**A**, *n* = 45) or did not have a late-life depression diagnosis (or cognitive impairment) (**B**, *n* = 38). Natural indirect and direct effects were estimated using structural equation modeling (R package *lavaan*). The total effect (c) represents the effect of synaptic density on plasma GFAP levels both directly (c’) and indirectly through the mediator (average causal mediated effect (ACME) = a*b). Effect sizes of scaled plasma biomarker concentrations (*z*-scores) are shown. Grey matter volume was normalized through division by the total intracranial volume and subsequently scaled through multiplication by 1,000 so that its magnitude was comparable to that of the other variables. Path weights and corresponding *P* values obtained through bootstrapping (*n* = 1,000) are superimposed.
